# The formation of mutated IgM memory B cells in rat splenic marginal zones is an antigen dependent process

**DOI:** 10.1371/journal.pone.0220933

**Published:** 2019-09-06

**Authors:** Jacobus Hendricks, Annie Visser, Peter M. Dammers, Johannes G. M. Burgerhof, Nicolaas A. Bos, Frans G. M. Kroese

**Affiliations:** 1 Department of Cell Biology, Immunology Section, University Medical Center Groningen, University of Groningen, Groningen, The Netherlands; 2 Institute for Life Science and Technology, Hanze University Groningen, Groningen, The Netherlands; 3 Department of Epidemiology, University Medical Center Groningen, University of Groningen, Groningen, The Netherlands; 4 Department of Rheumatology and Clinical Immunology, University Medical Center Groningen, University of Groningen, Groningen, The Netherlands; Stanford University School of Medicine, UNITED STATES

## Abstract

Previous studies in rodents have indicated that only a minor fraction of the immunoglobulin heavy chain variable region (IGHV-Cμ) transcripts carry somatic mutations and are considered memory B cells. This is in marked contrast to humans where nearly all marginal zone B (MZ-B) cells are mutated. Here we show in rats that the proportion of mutated IgM^+^ MZ-B cells varies significantly between the various IGHV genes analyzed, ranging from 27% mutated IGHV5 transcripts to 65% mutated IGHV4 transcripts. The observed data on mutated sequences in clonally-related B cells with a MZ-B cell or follicular B (FO-B) cell phenotype indicates that mutated IgM^+^ MZ-B and FO-B cells have a common origin. To further investigate the origin of mutated IgM^+^ MZ-B cells we determined whether mutations occurred in rearranged IGHV-Cμ transcripts using IGHV4 and IGHV5 genes from neonatal rat MZ-B cells and FO-B cells. We were not able to detect mutations in any of the IGHV4 and IGHV5 genes expressed by MZ-B cells or FO-B cells obtained from neonatal rat spleens. Germinal centres (GCs) are absent from neonatal rat spleen in the first few weeks of their life, and no mutations were found in any of the neonatal sequences, not even in the IGHV4 gene family which accumulates the highest number of mutated sequences (66%) in the adult rat. Therefore, these data do not support the notion that MZ-B cells in rats mutate their IGHV genes as part of their developmental program, but are consistent with the notion that mutated rat MZ-B cells require GCs for their generation. Our findings support that the splenic MZ of rats harbors a significant number of memory type IgM^+^ MZ-B cells with mutated IGHV genes and propose that these memory MZ-B cells are probably generated as a result of an antigen driven immune response in GCs, which still remains to be proven.

## Introduction

The splenic marginal zone (MZ) is a distinct anatomical compartment dominated by a unique population of B (MZ-B) lymphocytes, in addition to macrophages, dendritic cells in rodents and in humans also CD4^+^ T cells [1–3]. This compartment forms an interface between the splenic red and white pulp. This unique localization in combination with the blood flow through this compartment, allows intimate contact between antigens in the blood and cells in the MZ. MZ-B cells have a distinctive phenotype, generally characterized by high levels of IgM and low levels of IgD (IgM^high^IgD^low^). This contrasts with the dominant population of mature (naïve) follicular B (FO-B) cells located in the follicles of peripheral lymphoid organs, which express low levels of IgM and high levels of IgD (IgM^low^IgD^high^). MZ-B cells appear to be in a “pre-activated” state, which is illustrated for example by their high expression of CD80/CD86 and complement receptor 2 (CD21) on their membrane surface in comparison with FO-B cells [4]. MZ-B cells are primarily responsible for T cell-independent (TI) responses to polysaccharide antigens present on the surface of encapsulated bacteria [5, 6]. Another important role of MZ-B cells is facilitation of antigen transport towards the follicles [7]. MZ-B cells constitute a heterogeneous population of cells [8, 9]. The majority of MZ-B cells in rats and mice express unmutated transcripts for IgM heavy chain molecules and are considered to represent naïve B cells. On average their heavy chain complementarity determining region 3 (H-CDR3) is 2–3 amino acids shorter than their FO-B cell counterparts [10]. Autoantigens, rather than exogenous antigens are thought to play a role in the ligand selection of these naïve MZ-B cells [11, 12]. In addition to naïve B cells, a small fraction of the MZ-B cells are either unswitched or class-switched memory B cells as shown by immunization [13–18]. A hallmark of memory B cells is the presence of somatic mutations in the IGV genes [19]. Indeed, approximately 10–20% of rodent IgM^+^ MZ-B cells carry mutated IgM-encoding IGHV genes [10, 20]. Experimental data by Hendricks et al have revealed in rats the presence of class-switched B cells with a MZ-B cell phenotype, as defined by non-Ig markers, expressing somatically mutated IGHV genes encoding for IgG subclasses [21]. These class-switched memory MZ-B cells exhibited significantly fewer mutations, compared to memory B cells with a FO-B cell phenotype [21]. Their work also provided evidence to suggest that class-switched memory MZ-B cells and FO-B cells originate in a common germinal-center (GC). In contrast to rodents, nearly all MZ-B cells in human spleens express mutated IGHV genes [22, 23]. Phenotypically, these human B cells express CD27, which is an important, but not conclusive, characteristic property of human memory B cells [24]. Human MZ-B cells are therefore defined as IgM^+^IgD^+^CD27^+^ B cells [25]. The reason for the discrepancy between the frequency of mutated MZ-B cells in rodents and humans is not clear. It may result from developmental differences between the species. It has been proposed that, during development, mutations are introduced into the IGHV genes of MZ-B cells in an antigen-independent fashion to diversify the naïve Ig repertoire [26]. Methods of analysis of IGHV genes is another factor that contributes to the discrepancy between adult human and rodent MZ-B cells. Both in humans [22, 23] and rodents [10, 20] mutational analysis of IGHV genes derived from splenic MZ-B cells was carried out on a restricted set of IGHV genes of certain IGHV gene families. Whether these IGHV genes are representative of the mutation frequencies of IGHV genes of the entire MZ-B cell pool is not known. MZ-B cells are ligand selected and for this reason can result in significant differences in the frequencies of mutated IGHV genes between the individual IGHV genes or IGHV gene families in rodents and humans. This issue was addressed in the work described here by determining the mutation frequencies of individual IGHV genes that belong to several IGHV gene families that vary in size (viz. IGHV3, IGHV4, IGHV5) and are expressed in the rat MZ-B or FO-B cells. Whether the mutated MZ-B cells in humans represent bona fide memory B cells is a matter of debate. On the one hand, these B cells are considered true memory cells, generated in GCs during antigen-driven humoral immune responses [27, 28]. On the other hand, it has been proposed that these cells are generated during TI immune responses [29]. Weller and co-workers argued that the presence of mutations reflects an intrinsic property of MZ-B cells that is exploited by these cells to diversify their primary antibody repertoire [25, 26, 30]. According to Weller and colleagues, the IgM^+^IgD^+^CD27^+^ MZ-B cells are *not* memory cells, but cells that develop in the absence of antigen, in a TI fashion, outside the GCs, along a pathway that differs from classical memory B cells. This conclusion was initially based on the finding that patients with CD40L or CD40 deficiency harbor mutated IgM^+^IgD^+^CD27^+^ MZ-B cells in the blood [25, 30]. These patients lack the classical cognate T-B cell collaboration that is required for the development of GCs. In support of the hypothesis of Weller, the majority of blood and splenic MZ-B cells in young children under the age of 2 years are mutated with no sign of antigen-driven clonal expansion [25, 26]. Scheeren et al. [31] also observed that a low fraction (~20%) of human fetal splenic IgM^+^IgD^+^CD27^+^ B cells are mutated, and these authors hypothesized that somatic hypermutation (SHM) in this population occurs mainly during foetal development and in very young children. MZ-B cells are already found early during ontogeny in the rat spleen [11, 32]. At that time, GCs are absent from spleen [33, 34]. Whether early in neonatal life the IGHV genes expressed by MZ-B cells in rodents are mutated or not is not known and was studied here. We show that, in contrast to humans, neonatal MZ-B cells in rats are all unmutated, supporting the view that, at least in rodents, a significant number of adult IgM^+^ mutated MZ-B cells are memory B cells that can be considered to be formed in GCs.

## Materials and methods

### Animals

Adult male at 4.5 months of age and pregnant BN/SsNOlaHsd rats were obtained from Harlan (Horst, The Netherlands). Rats were housed under clean, conventional conditions at the Central Animal Facility of the University Medical Center Groningen. The adult male rats were housed until the age of 9 months. Two-day-old neonatal rats of both sexes were killed by decapitation. All experiments were approved by the Animal Ethics Committee of the University of Groningen.

### Isolation and purification of B cell subsets by Flow cytometry

Rat B-lymphocytes were isolated and purified from splenic tissue as described previously [11, 35]. Briefly, spleen cell suspensions from 2 adult male rats and from 5 pooled spleens of two day old neonatal rats were separately prepared and labeled for flow-cytometry with the following mouse monoclonal antibodies: FITC conjugated anti-rat IgM (HIS40; eBioscience, San Diego, CA, USA), biotinylated anti-rat IgD (MaRD3; AbD Serotec, Oxford, UK), APC conjugated anti-rat CD90/Thy1.1 (HIS51; eBioscience) and PE-conjugated anti-rat TCRαβ (R73; eBioscience); TCRγδ (V65; eBioscience), CD161a/NKRP1a (10/78; BD Pharmingen). Biotinylated mAb were revealed with streptavidin conjugated to the tandem fluorochrome PE-Cy5.5 (Ebioscience). The PE channel was used as a “dump” channel: only PE cells negative (i.e. Dump^-^ and CD90^-^ cells) were sorted. Herewith, we were able to exclude immature B cells (i.e. CD90^+^ B-cells: [11, 35], T cells and NK cells from our sorts. Cell analysis and cell sortings were performed with a MoFlo flow cytometer (Cytomation, Ft Collins, CO). Dead cell, plasma cell, monocyte/macrophage, and erythrocyte contamination was excluded from sorting by using forward and side scatter profiles. Sorted FO-B cells (CD90^-^IgD^high^IgM^low^) and MZ-B cells (CD90^-^IgM^high^IgD^low^) were collected in sterile FACS tubes (Greiner Bio-One, Alphen a/d Rijn, The Netherlands) containing 500 μl newborn calf serum (PAA laboratories GmbH, Pasching, Austria). At least one million cells per B cell subset were sorted. B cell subsets were obtained with > 95% purity. FlowJo software (Tree Star, San Carlos, CA) was used for flow cytometry data analysis.

### Molecular cloning of IGHV-Cμ transcripts

Total RNA was extracted from sorted cells using the Absolutely RNA Miniprep kit (Stratagene, La Jolla, CA, USA) according to instructions of the manufacturer. Briefly, sorted cells were pelleted by 300xg centrifugation for 10 min at 4°C and then resuspended in a total volume of 350μl lysis buffer containing β-mercaptoethanol (Stratagene). First strand cDNA was synthesized using an oligo-(dT)12-18 primer (Invitrogen, Breda, The Netherlands) and SuperScriptTMII reverse transcriptase (200U/μl; Invitrogen) as described in the manufacturer’s protocol. Rearranged IGHV3-Cμ, IGHV4-Cμ IGHV5-Cμ transcripts were amplified in a 50μl reaction mixture, containing 2μl cDNA of either IGHV3-Cμ, IGHV4-Cμ or IGHV5-Cμ family-specific primer, plus 0.6 pmol/μl universal Cμ constant region primer and 2.5U Taq DNA Polymerase (Invitrogen). The IGHV gene family specific primers were: IGHV3:5'-TGAAACCCTCACAGTCACTC-3', IGHV4:5’-GGTGCA**R**CCTGGAAGATCCT-3' and IGHV5'-CTTAGTGCAGCCTGGAAGGT-3' [10]. Individual IGHV gene family specific primers were used in separate RT-PCR reactions in combination with the constant region Cμ primer 5’-CAACACTGAAGTCATCCAGGG-3’. To assess the amount and quality of the cDNA, PCR was also performed for β-actin, using β-actin-specific primers as described by Stoel et al. [36]. The PCR program for amplification of IGHV-Cμ transcripts and β-actin consisted of 35 cycles of 30 sec at 94°C (2 min in the first cycle), 1 min at 58°C and 1 min at 72°C, respectively. This program was followed by an additional incubation period of 25 min at 72°C to allow extension of all IGHV-Cμ products. The quality and size of the PCR products were evaluated by agarose gel electrophoresis.

### Cloning and sequencing

PCR products were cloned into the pJET1/blunt vectors using the GeneJET^TM^ PCR Cloning Kit (Fermentas Life Sciences). TOP10F E. coli competent cells (Invitrogen) were transformed with plasmids containing the PCR product. Plasmid DNA was isolated from randomly picked colonies with the Nucleospin Plasmid QuickPure kit (Bioke, Leiden, The Netherlands). Size of the inserts was determined by digestion of a DNA sample of the plasmids with the appropriate restriction enzymes followed by agarose gel electrophoresis. Plasmids containing an insert of approximately 500 bp were sequenced in both directions by ServiceXS (Leiden, The Netherlands). Sequence processing was performed using EMBL/Genbank Data Libraries and Chromas software (Digital River GmbH, Cologne, Germany). IGHV-Cμ sequences displaying 100% identity and obtained from the same PCR amplification, might be derived from a single B cell and were therefore counted only once in our subsequent analysis. The presence of SHM in IGHV genes are another hallmark of memory B cells and therefore mutated IGHV-Cμ transcripts identified with >2 number of mutations in the VDJ region were considered to be IgM^+^ memory B cells. Mutational analysis was performed using IMGT/V QUEST database (www.imgt.org/IMGT_vquest/share/textes/). We have calculated the mutations (>2 in the VDJ region) of H-CDR and H-FR regions from IGHV-Cμ transcripts as previously reported by Hendricks et al. [21].

### Statistical analysis of IGHVDJ–Cμ transcripts

Statistical analysis of the sequence data was performed using SPSS 16 software (SPSS Inc. Chicago, Ill. USA). IGHV-Cμ sequences displaying 100% identity were considered to be derived from a single B cell and counted only once for statistical analysis. Since Taq DNA polymerase errors might be responsible for 1–2 mutations per sequence, we considered only sequences with more than 2 mutations as truly mutated [10]. The number of mutations was determined by counting the number of nucleotide mismatches in comparison with each IGHV gene sequence to its closest germline counterpart. We used Fisher’s exact test to determine possible differences between groups with regard to the binary response variable indicating mutation or not. Non-parametric tests, Kruskal-Wallis and Mann-Whitney, were used to compare groups with respect to the number of mutations. In all statistical tests, a p-value < 0.05 was considered significant.

## Results

### Analysis of the adult rat IGHV genes in FO-B cells and MZ-B cells

**Fig 1:**
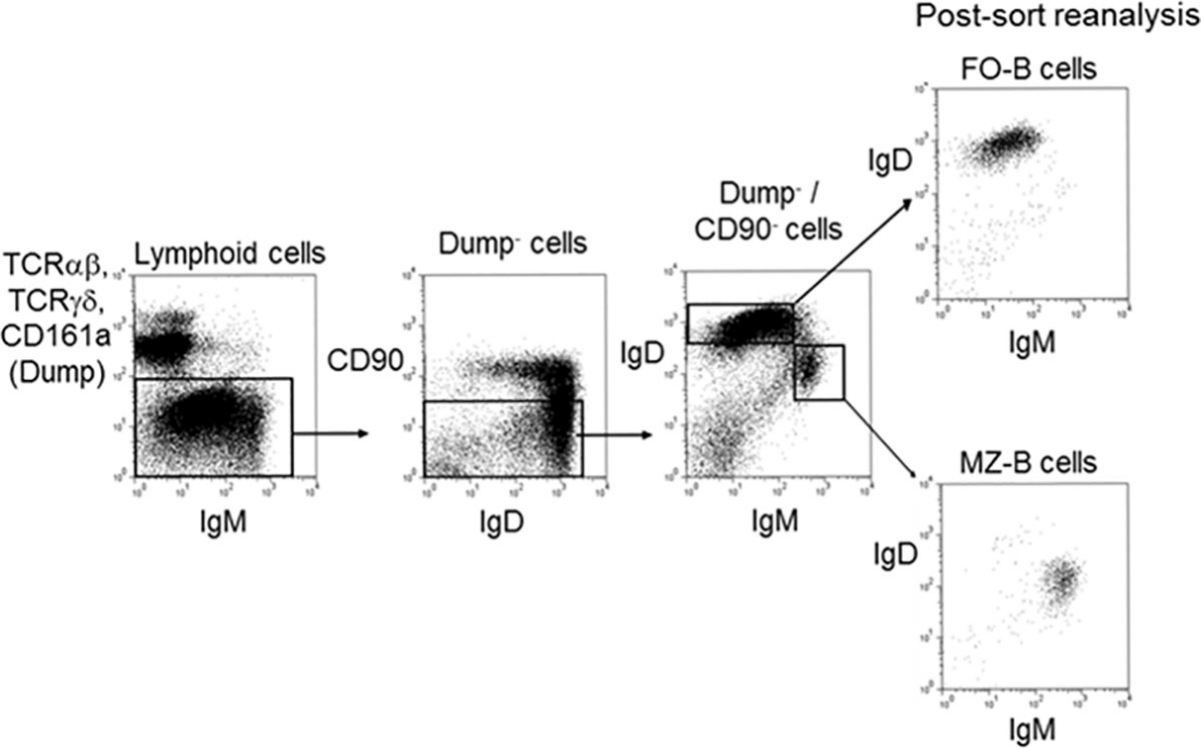
Four colour cytometry of FO-B cells and MZ-B cells. Single cell suspensions of spleen from rats were stained with FITC conjugated anti-rat IgM, biotinylated anti-rat IgD, APC conjugated anti-rat CD90/Thy1.1 and PE-conjugated anti-rat TCRαβ, TCRγδ, CD161a/NKRa. Biotinylated monoclonal antibodies were revealed with streptavidin conjugated to the tandem fluorochrome PE-Cy5.5. Viable lymphocytes were gated by forward scatter and side scatter profiles. Acquisition gates were set to exclude PE positive cells (T cells and NK cells) and CD90 positive (immature) B cells. Mature FO-B cells, defined as CD90^-^IgD^high^IgM^low^ and MZ-B defined as CD90^-^IgM^high^IgD^low^were sorted. Post sort reanalysis showed that the purity of FO-B cells and MZ-B cells was >95%.

Recently, we have constructed and annotated the complete genomic repertoire of the IGH locus of the BN rat [37]. The completion of the IGH locus has allowed us to analyze individual IGHV genes among different IGHV gene families by FO-B and MZ-B cells. In (Fig 1), the strategy for sorting of FO-B and MZ-B cells is illustrated. Viable lymphocytes were gated on the basis of forward-sideward scatter profiles, and non-T, non-NK cells (Dump^-^ cells) were further analyzed. FO-B cells and MZ-B cells were subsequently defined as CD90^-^IgM^low^IgD^high^ and CD90^-^IgM^high^IgD^low^. The post sort purity for FO-B cells and MZ-B cells was >95%.

Expressed IGHV3, IGHV4 and IGHV5 genes from adult rats were amplified, cloned and sequenced (Table 1). These IGHV gene families were chosen because of their difference in size, with respectively 4, 2 and 26 potentially functional IGHV genes in the BN rat. We obtained 19 and 17 complete IGHV3-Cμ transcripts from FO-B cells and MZ-B cells, respectively.

**Table 1:** Sequence analysis of IGHV3,4 and 5-Cμ transcripts expressed by FO-B cells and MZ-B cells from adult BN rat spleen.

**Clone**^**a**^	**IGHV**	**IGHD**	**IGHJ**	**Mutations**^**b**^	**H-CDR3**^**c**^	
** **	**member**	**member**	**member**	** **	**N**^**f**^	**Amino acids**
**Sequences of IGHV3 gene family from FO-B**^**d**^ **cells**						
A2RFV3-A4	IGHV3S1	IGHD1-7	IGHJ1	0	12	ARTTRVYWYFDF
A2RFV3-B2	IGHV3S1	IGHD4-4	IGHJ2	0	9	ARFVGYFDY
A2RFV3-B3	IGHV3S1	IGHD4-1	IGHJ2	1	11	AYSPGGYRFDY
A2RFV3-C2	IGHV3S1	IGHD1-6	IGHJ2	0	12	ARYGATEGIVDY
A2RFV3-D1	IGHV3S1	IGHD1-7	IGHJ1	0	16	ARFYDGSYYYDWYFDF
A2RFV3-D3	IGHV3S1	IGHD1-7	IGHJ3	0	17	ARYYGIYYYSSYDWFAY
A2RFV3-E1	IGHV3S1	IGHD1-8	IGHJ3	0	17	ARAGGRDSYAHVGWFAY
A2RFV3-E2	IGHV3S1	IGHD1-3	IGHJ2	1	11	ARLYSIAAPYY
A2RFV3-E3	IGHV3S1	IGHD4-1	IGHJ3	0	8	ARNPGFAY
A2RFV3-G3	IGHV3S1	IGHD3-3	IGHJ2	2	11	ARSGQKSLFDY
A2RFV3-H1	IGHV3S1	IGHD1-7	IGHJ2	0	16	ARYGDYYDGSYYAFDY
A2RFV3-H3	IGHV3S1	IGHD3-1	IGHJ2	0	10	ASPYPGQRWY
A2RFV3-A3	IGHV3S3	IGHD1-3	IGHJ2	1	12	ARSELQWYYFDY
A2RFV3-B4	IGHV3S3	IGHD5-1	IGHJ2	0	16	ARRPITGSGGGYYFDY
A2RFV3-F2	IGHV3S3	IGHD5-1	IGHJ4	0	12	ASGNWDSYVMDA
A2RFV3-G1	IGHV3S3	IGHD1-7	IGHJ2	0	15	ARSSYYYDGSYSLDY
A2RFV3-A1	IGHV3S5	IGHD1-5	IGHJ3	0	10	AGNNLDWFAY
A2RFV3-F3	IGHV3S5	IGHD1-8	IGHJ4	1	16	ASPLDGYYPYYYVMDA
**Sequences of IGHV3 gene family from MZ-B**^**e**^ **cells**						
A2MZV3-A5	IGHV3S1	IGHD1-1	IGHJ2	0	9	ARRTVSFDY
A2MZV3-A6	IGHV3S1	IGHD1-4	IGHJ2	8	12	ARRDPGITLFDY
A2MZV3-B7^g^	IGHV3S1	IGHD1-3	IGHJ2	0	13	ARGQQLSEYYFDY^C#1^
A2MZV3-F5^g^	IGHV3S1	IGHD1-3	IGHJ2	1	13	ARGQQLSEYYFDY^C#1^
A2MZV3-C5	IGHV3S1	IGHD1-7	IGHJ1	0	14	ARYDGSYYYWYFDF
A2MZV3-C7^h^	IGHV3S1	IGHD1-4	IGHJ2	1	12	ARSGGYNYYFDY^C#2^
A2MZV3-E7^h^	IGHV3S1	IGHD1-4	IGHJ2	0	12	ARSGGYNYYFDY^C#2^
A2MZV3-D4	IGHV3S1	IGHD1-6	IGHJ3	5	10	ARYSERGFAY
A2MZV3-E4	IGHV3S1	IGHD1-5	IGHJ2	0	12	ARGGIYNTYFDY
**Clone**^**a**^	**IGHV**	**IGHD**	**IGHJ**	**Mutations**^**b**^	**H-CDR3**^**c**^	
** **	**member**	**member**	**member**	** **	**N**^**f**^	**Amino acids**
A2MZV3-E5	IGHV3S1	IGHD4-1	IGHJ2	7	13	ARKGDSNSGLFDY
A2MZV3-F6	IGHV3S1	IGHD1-1	IGHJ2	0	15	ARGGVYYGLLSSFDY
A2MZV3-G6	IGHV3S1	IGHD1-7	IGHJ2	0	13	ARSTTVVHYYFDY
A2MZV3-G7	IGHV3S1	IGHD1-1	IGHJ2	4	12	ARSGYTTDYPDY
A2MZV3-H4	IGHV3S1	IGHD3-2	IGHJ2	0	9	ARSTDYFDY
A2MZV3-E6	IGHV3S3	IGHD3-1	IGHJ2	0	10	ARSGSGDFDY
A2MZV3-B5	IGHV3S5	IGHD1-5	IGHJ4	4	12	ARRTTSDYVMDA
**Sequences of IGHV4 gene family from FO-B**^d^ cells						
A2RFV4-2^i^	IGHV4S2	IGHD4-1	IGHJ2	9	9	VREAFGVRE^C#3^
A2RFV4-2.27	IGHV4S2	IGHD1-2	IGHJ1	0	10	GGSLYWYFDF
A2RFV4-5	IGHV4S2	No IGHD	IGHJ3	0	5	ASRAY
A2RFV4-6	IGHV4S2	IGHD1-6	IGHJ4	4	11	TRAGTVLQMDA
A3RFV4-12	IGHV4S2	IGHD1-8	IGHJ2	0	12	ARASYYDGYGDY
A3RFV4-3^j^	IGHV4S2	IGHD4-1	IGHJ2	11	9	VREAFGVDY^C#4^
A3RFV4-3.2	IGHV4S2	IGHD1-4	IGHJ3	0	13	ARADGYNFNWFAY
A3RFV4-3.4	IGHV4S2	IGHD3-3	IGHJ1	0	12	ARLWRRYWYFDF
A3RFV4-43	IGHV4S2	IGHD1-5	IGHJ2	1	12	ARWNNYDYYFDY
A3RFV4-9	IGHV4S2	IGHD1-5	IGHJ2	0	11	AREDYNNIGDH
A2MZV4-1^i^	IGHV4S2	IGHD4-1	IGHJ2	9	9	VREAFGVRE^C#3^
A2MZV4-10	IGHV4S2	IGHD1-6	IGHJ2	1	9	AREVGYFDY
A2MZV4-11	IGHV4S2	IGHD3-4	IGHJ2	1	9	TRARKSVDY
A2MZV4-12	IGHV4S2	IGHD1-1	IGHJ3	2	6	EGGIIG
A2MZV4-13	IGHV4S2	IGHD1-6	IGHJ4	11	11	ARASGQRVLDA
A2MZV4-14^k^	IGHV4S2	IGHD4-1	IGHJ4	5	14	TRREFGPHYYVMDA^C#5^
A2MZV4-3^k^	IGHV4S2	IGHD4-1	IGHJ4	7	14	TRREFGPHYYVMDA^C#5^
A2MZV4-2.1	IGHV4S2	IGHD1-1	IGHJ3	9	12	ARGLYYGFGFAY
A2MZV4-2.10	IGHV4S2	IGHD4-1	IGHJ4	0	13	ARARNSDYYVMDA
A2MZV4-2.11	IGHV4S2	IGHD4-1	IGHJ2	0	10	ASHERYTSDY
A2MZV4-2.13^l^	IGHV4S2	IGHD4-2	IGHJ2	11	9	VREHFGVDF^C#6^
A2MZV4-2.17^l^	IGHV4S2	IGHD4-2	IGHJ2	13	9	VREHFGVDF^C#6^
A2MZV4-2.14	IGHV4S2	IGHD4-1	IGHJ2	8	9	AREAFGVRE
A2MZV4-2.18	IGHV4S2	IGHD1-6	IGHJ2	10	9	AREEAGIDY
A2MZV4-2.20	IGHV4S2	IGHD1-6	IGHJ4	5	9	VREALGVNA
A2MZV4-2.21	IGHV4S2	IGHD2-2	IGHJ2	9	9	VREAYGVDY
A2MZV4-2.3	IGHV4S2	IGHD1-1	IGHJ2	1	25	AREGVYYYSSYRDVYYGLLPGYFDY
A2MZV4-2.4	IGHV4S2	IGHD1-7	IGHJ2	8	15	ARGYYYDGSYYHFDY
A2MZV4-2.7	IGHV4S2	IGHD1-5	IGHJ1	2	16	AREALITTTSYWYFDF
A2MZV4-2.8	IGHV4S2	IGHD1-6	IGHJ4	16	9	VREALGVDA
A2MZV4-7^m^	IGHV4S2	IGHD1-1	IGHJ2	4	13	ARARGMSTTDYLY^C#7^
**Clone**^**a**^	**IGHV**	**IGHD**	**IGHJ**	**Mutations**^**b**^	**H-CDR3**^**c**^	
** **	**member**	**member**	**member**	** **	**N**^**f**^	**Amino acids**
A2MZV4-5	IGHV4S2	IGHD4-2	IGHJ2	6	9	VREELGVDY
A2MZV4-8	IGHV4S2	IGHD5-1	IGHJ1	10	13	GRLSWELYWYFDF
A2MZV4-9	IGHV4S2	IGHD3-2	IGHJ2	3	9	VRAHSSAGD
A3MZV4-1	IGHV4S2	IGHD1-4	IGHJ2	2	15	ARGTSYGSSSDYFDY
A3MZV4-10	IGHV4S2	IGHD1-3	IGHJ2	0	15	ARALDYYSSYIYLDY
A3MZV4-35	IGHV4S2	IGHD1-6	IGHJ2	13	12	ARGDYYRGDFDY
A3MZV4-11^n^	IGHV4S2	IGHD1-6	IGHJ2	11	9	VREHLGVDY^C#8^
A3MZV4-3.1^n^	IGHV4S2	IGHD1-6	IGHJ2	11	9	VREHLGVDY^C#8^
A3MZV4-36	IGHV4S2	IGHD1-3	IGHJ2	0	11	AREDYSGDFDY
A3MZV4-14	IGHV4S2	IGHD1-8	IGHJ2	5	10	SGGLGWIFDY
A3MZV4-15	IGHV4S2	IGHD1-1	IGHJ4	0	17	ARVLFMYTTDYQGVMDA
A3MZV4-16	IGHV4S2	IGHD4-1	IGHJ2	19	9	VREDFGVDY
A3MZV4-17	IGHV4S2	IGHD5-1	IGHJ2	0	11	ARARETGNFDY
A3MZV4-18	IGHV4S2	IGHD1-2	IGHJ2	9	15	TRGPSYGSDSDFFDY
A3MZV4-19	IGHV4S2	IGHD1-4	IGHJ2	8	15	ARGTSYGSNSDYFDY
A3MZV4-20	IGHV4S2	IGHD4-1	IGHJ2	8	9	AREAFGVDY
A3MZV4-20B^o^	IGHV4S2	IGHD1-4	IGHJ2	14	10	AKSGPGIIEY^C#9^
A3MZV4-7^o^	IGHV4S2	IGHD1-4	IGHJ2	13	10	AKSGPGIIEY^C#9^
A3MZV4-22	IGHV4S2	IGHD4-1	IGHJ2	9	9	IREAFGVDY
A3MZV4-37	IGHV4S2	IGHD3-2	IGHJ1	15	14	AGLRSGAPYWYLDF
A3MZV4-23	IGHV4S2	IGHD1-6	IGHJ3	9	12	ARELSTGEWFAY
A3MZV4-29	IGHV4S2	IGHD1-7	IGHJ2	2	14	ARSLMVVISHYFDY
A3MZV4-3	IGHV4S2	IGHD1-6	IGHJ4	0	10	ARRRSDVMDA
A3MZV4-3.12	IGHV4S2	IGHD1-6	IGHJ4	0	14	ARVGDSSYYYVMDA
A3MZV4-3.14	IGHV4S2	IGHD1-6	IGHJ3	1	11	VRERSTEGFAY
A3MZV4-3.2^j^	IGHV4S2	IGHD4-1	IGHJ2	14	9	VREAFGVDY^C#4^
A3MZV4-3.5	IGHV4S2	IGHD4-1	IGHJ2	12	9	VREDLGVDY
A3MZV4-30	IGHV4S2	IGHD2-2	IGHJ2	14	9	AREIPPVDY
A3MZV4-31	IGHV4S2	IGHD1-4	IGHJ4	11	11	ARAVISRVLDA
A3MZV4-32	IGHV4S2	IGHD4-1	IGHJ2	6	9	VREEFGVDY
A3MZV4-5N	IGHV4S2	IGHD1-6	IGHJ2	11	9	VREQRGVDY^C15^
A3MZV4-5A	IGHV4S2	IGHD1-2	IGHJ2	8	15	TRGPSYGSDSDYFDY
A3MZV4-9	IGHV4S2	IGHD1-5	IGHJ2	0	11	ARADNNSGFDY
**Sequences of IGHV5 genes from FO-B**^**d**^ **cells**						
A2RFV5-39	IGHV5S16	IGHD1-1	IGHJ3	0	12	ARPNYYSGPLAY
A3RFV5-11	IGHV5S13	IGHD1-2	IGHJ2	0	9	ARRAMGFDY
A2RFV5-42^p^	IGHV5-1	IGHD1-6	IGHJ2	17	9	TKGVGGPDY^C#10^
A2RFV5-46	IGHV5S10	IGHD5-1	IGHJ2	0	9	ATHLGYFDY
A2RFV5-38	IGHV5S14	IGHD1-1	IGHJ2	1	12	VRLCGERDYFDY
**Clone**^**a**^	**IGHV**	**IGHD**	**IGHJ**	**Mutations**^**b**^	**H-CDR3**^**c**^	
** **	**member**	**member**	**member**	** **	**N**^**f**^	**Amino acids**
A2RFV5-45	IGHV5S14	IGHD1-6	IGHJ1	1	16	ARHVPLHYGGHGYFDF
A3RFV5-14N	IGHV5S14	IGHD1-2	IGHJ2	0	8	ARRDDFDY
A3RFV5-48	IGHV5S14	IGHD1-6	IGHJ1	0	17	ARLPAYYGGYSELPFAY
A3RFV5-5	IGHV5S14	IGHD1-1	IGHJ2	0	18	ARHLMYTTDYYYPGAFDY
A2RFV5-17	IGHV5S23	IGHD1-4	IGHJ3	4	14	ARGDYPGITGWFAY
A2RFV5-19	IGHV5S23	IGHD1-4	IGHJ2	0	6	ARPYSV
A3RFV5-12	IGHV5S23	IGHD1-8	IGHJ1	1	20	ARPPRWDYDGYYHVGWYFDF
A3RFV5-15	IGHV5S23	IGHD1-1	IGHJ4	6	17	ARSLMYTTDYYYGVMDA
A3RFV5-8	IGHV5S23	IGHD1-7	IGHJ2	4	12	ARGDDGSYYFDY
A3RFV5-3	IGHV5S27	IGHD1-4	IGHJ2	4	12	ARRPPGYNPFDY
A3RFV5-45	IGHV5S27	IGHD1-7	IGHJ3	0	20	ARHGADGAMMVVITNGWFAY
A3RFV5-47	IGHV5S29	IGHD2-3	IGHJ2	2	10	TTDRLSTFDY
A2RFV5-22	IGHV5S30	IGHD1-1	IGHJ3	4	17	ARHMYTTDYYHGDWFAY
A2RFV5-23	IGHV5S30	IGHD1-4	IGHJ3	2	14	ATRPLPGYNYGFAY
A2RFV5-35	IGHV5S30	IGHD1-8	IGHJ3	11	9	ARQDQEFAY
A2RFV5-37	IGHV5S30	IGHD1-7	IGHJ2	4	13	ARLDYYDGSYYDY
A2RFV5-42	IGHV5S30	IGHD4-1	IGHJ2	0	9	ATVAGYFDY
A2RFV5-8	IGHV5S30	IGHD1-3	IGHJ2	0	8	ATLLYSGH
A3RFV5-13N	IGHV5S30	IGHD1-1	IGHJ3	0	16	ATDSPTTDYYSNWFAY
A3RFV5-2	IGHV5S30	IGHD1-6	IGHJ3	0	17	ATDTDYGGYSELGGFAY
A3RFV5-4	IGHV5S43	IGHD4-2	IGHJ3	1	13	TRDRGYSSHWFAY
A3RFV5-46	IGHV5S43	IGHD1-3	IGHJ4	0	14	TREPGDYSSYVMDA
A3RFV5-13	IGHV5S43	IGHD1-7	IGHJ2	2	13	TRVGHYYSSYFDY
A2RFV5-18	IGHV5S45	IGHD1-1	IGHJ2	2	12	ARRYTTDYWFDY
A2RFV5-41	IGHV5S45	IGHD1-2	IGHJ2	0	10	ARPPYGAFDY
A3RFV5-43	IGHV5S45	IGHD1-1	IGHJ3	4	24	TTGAYSSYAVMYTTDYYYAGWFAY
A3RFV5-42	IGHV5S45	IGHD2-2	IGHJ1	2	12	ARRDTLYWYFDF
A2RFV5-47	IGHV5S57	IGHD3-3	IGHJ1	1	15	TRASSSYVSDWYFDF
A3RFV5-44	IGHV5S57	IGHD1-2	IGHJ2	0	11	TRTRVSYYFDY
A2RFV5-43	IGHV5S65	IGHD1-5	IGHJ2	1	13	AKDQGNNYGYFDY
A3RFV5-2	IGHV5S74	IGHD5-1	IGHJ2	2	11	ARGHGDYYFDY
**Sequences of IGHV5 genes from MZ-B**^**e**^ **cells**						
A2MZV5-37^p^	IGHV5-1	IGHD1-6	IGHJ2	13	9	TKGVGGPDY^C#10^
A2MZV5-39	IGHV5S10	IGHD4-1	IGHJ2	0	11	ATHPGEYYFDY
A3MZV5-4	IGHV5-1	IGHD1-6	IGHJ2	16	9	AKGVGGPDY
A2MZV5-2.5	IGHV5S13	IGHD1-1	IGHJ2	0	14	ARFGLITVAVHFDY
A2MZV5-20	IGHV5S13	IGHD1-6	IGHJ1	1	18	ARTTGLTTEGIGYWYFDF
A2MZV5-38	IGHV5S13	IGHD1-1	IGHJ3	4	7	GYYGFAY
A3MZV5-16	IGHV5S13	IGHD1-6	IGHJ3	2	14	ARHETTVVTGWFAY
**Clone**^**a**^	**IGHV**	**IGHD**	**IGHJ**	**Mutations**^**b**^	**H-CDR3**^**c**^	
** **	**member**	**member**	**member**	** **	**N**^**f**^	**Amino acids**
A3MZV5-38	IGHV5S13	IGHD1-1	IGHJ2	1	11	ASIITTGYFDY
A3MZV5-66	IGHV5S13	IGHD1-3	IGHJ1	1	12	ASQSSYNWYFDF
A2MZV5-11	IGHV5S14	IGHD1-1	IGHJ2	0	9	ARRLLQWDY
A2MZV5-33	IGHV5S14	IGHD1-4	IGHJ2	1	17	ARGGINNIGTTRGVMDA
A3MZV5-3.8	IGHV5S14	IGHD1-7	IGHJ2	0	12	ARYYYDGPWGDY
A3MZV5-5	IGHV5S14	IGHD1-7	IGHJ2	2	15	ARTGFYYYSGDYFDY
A3MZV5-8	IGHV5S14	IGHD1-7	IGHJ2	1	13	ARHYYDGSYYFDY
A2MZV5-14	IGHV5S16	IGHD5-1	IGHJ2	4	7	TTDLNNY
A2MZV5-19	IGHV5S16	IGHD1-8	IGHJ1	0	11	ATCSPYWYFDF
A2MZV5-22	IGHV5S16	IGHD1-7	IGHJ4	4	11	ATDEGGGVMDA
A3MZV5-13	IGHV5S16	IGHD1-6	IGHJ3	4	12	TTLYGGPPWFAY
A2MZV5-21	IGHV5S23	IGHD1-2	IGHJ1	5	15	ARQSTYYEDGWYFDF
A3MZV5-3.3	IGHV5S23	IGHD1-2	IGHJ3	4	14	ATEGTMGMSDWFAY
A2MZV5-23	IGHV5S27	IGHD1-4	IGHJ3	0	13	ARPYGYNYRWFAY
A3MZV5-61	IGHV5S29	IGHD1-6	IGHJ3	0	12	TTDRGNYGWFAY
A3MZV5-65	IGHV5S29	IGHD1-7	IGHJ2	1	13	TSPLTTVVPYFDY
A2MZV5-18	IGHV5S30	IGHD1-5	IGHJ2	1	13	ARHDNNYVAYFDY
A2MZV5-2.2	IGHV5S30	IGHD1-3	IGHJ2	2	17	ATDQYYSSYTLAGYFDY
A3MZV5.57	IGHV5S30	IGHD1-3	IGHJ3	1	15	ATDRAYRSYIPTFAY
A3MZV5-10	IGHV5S30	IGHD1-6	IGHJ1	0	12	ATEIDSDWYFDF
A3MZV5-14	IGHV5S30	IGHD1-8	IGHJ2	0	6	ATLSYY
A3MZV5-17	IGHV5S30	IGHD1-7	IGHJ2	5	15	AKMWGGSYYYVPFDY
A3MZV5-2N	IGHV5S30	IGHD4-1	IGHJ2	0	6	ATDSSG
A3MZV5-24	IGHV5S30	IGHD5-1	IGHJ3	0	11	ATDDQLDWFAY
A3MZV5-25	IGHV5S30	IGHD4-1	IGHJ3	11	11	AHNAGDVWFPY
A3MZV5-26	IGHV5S30	IGHD1-3	IGHJ2	0	13	ATGVHYSSYIFDY
A3MZV5-3.11	IGHV5S30	IGHD1-2	IGHJ2	2	10	ATQLGGSFDY
A3MZV5-3.4	IGHV5S30	IGHD1-8	IGHJ2	1	12	ATGDYYDGYPDY
A3MZV5-3.6	IGHV5S30	IGHD1-7	IGHJ2	0	13	ATDRSDDGGFFDY
A3MZV5-3.7	IGHV5S30	IGHD1-1	IGHJ2	0	12	ATDHVYYGLLGA
A3MZV5-33	IGHV5S30	IGHD1-1	IGHJ3	0	14	ATAGDTTDYSRFAY
A3MZV5-39	IGHV5S30	IGHD1-6	IGHJ2	0	11	ARGINYGGYAH
A3MZV5-6	IGHV5S30	IGHD1-1	IGHJ3	2	14	ATEVYYGLSDWFAY
A3MZV5-63	IGHV5S30	IGHD1-4	IGHJ2	1	11	ATDEAGDTGDY
A3MZV5-8	IGHV5S30	IGHD1-5	IGHJ2	0	12	ATAFITTTGFDY
A2MZV5-4	IGHV5S30	IGHD1-7	IGHJ3	0	13	ATDGGYAPRWFAY
A2MZV5-25	IGHV5S45	IGHD1-4	IGHJ2	2	10	TTGDMGITPY
A2MZV5-35	IGHV5S45	IGHD1-2	IGHJ4	2	11	ARQGDYGPMDA
A2MZV5-9	IGHV5S45	IGHD4-2	IGHJ1	1	13	ARRGGSAYWYFDF
**Clone**^**a**^	**IGHV**	**IGHD**	**IGHJ**	**Mutations**^**b**^	**H-CDR3**^**c**^	
** **	**member**	**member**	**member**	** **	**N**^f^	**Amino acids**
A3MZV5-11	IGHV5S32	IGHD1-1	IGHJ4	4	17	ATDGAFTTNYFYDVMAA
A3MZV5-12	IGHV5S32	IGHD1-6	IGHJ2	1	12	ARQGYGGYPFDY
A2MZV5-36	IGHV5S36	IGHD1-6	IGHJ2	7	11	TTEVLQWVFDY
A2MZV5-40	IGHV5S36	IGHD1-3	IGHJ3	1	12	TTGTIAANWFAY
A3MZV5-21	IGHV5S36	IGHD1-2	IGHJ2	6	7	ATGLGDY
A2MZV5-12	IGHV5S43	IGHD1-4	IGHJ2	0	13	TREGPYGYNYFDY
A3MZV5-3.15	IGHV5S43	IGHD1-1	IGHJ4	14	10	TIYSNYVMDA
A3MZV5-6	IGHV5S43	IGHD1-6	IGHJ3	0	9	TRGTTEAAY
A3MZV5-10	IGHV5S65	IGHD1-2	IGHJ2	0	9	AKESTMGMG
A3MZV5-36	IGHV5S65	IGHD1-1	IGHJ2	19	7	AINKYNY
A3MZV5-7	IGHV5S65	IGHD1-2	IGHJ2	6	13	AKDSYGGYRYFDY

^a^Cμ (IgM) transcripts from FO-B cells and MZ-B cells

^b^Mutations, nucleotide differences between IMGT germline gene and rearranged Cμ transcript

^c^H-CDR3, heavy chain complementarity determining region 3

^d^FO-B cells, recirculating follicular B cells

^e^MZ-B cells, marginal zone B cells

^f^Lenght of H-CDR3 in amino acids

^g^The sequence A2MZV3-B7 and A2MZV3-F5 are from clonally related B cells and designated as clone set ^C#1^

^h^The sequence A2MZV3-C7 and A2MZV3-E7 are from clonally related B cells and designated as clone set ^C#2^

^i^The sequences A2RFV4-2 and A2MZV4-1 are from clonally related B cells and designated as clone set ^C#3^

^j^The sequences A3RFV4-3 and A3MZV4-3.2 are from clonally related B cells and designated as clone set ^C#4^

^k^The sequences A2MZV4-14 and A2MZV4-3 are from clonally related B cells and designated as clone set ^C#5^

^l^The sequences A2MZV4-2.13 and A2MZV4-2.17 are from clonally related B cells and designated as clone set ^C#6^

^m^The sequences A2MZV4-2.9 and A2MZV4-7 are from clonally related B cells and designated as clone set ^C#7^

^n^The sequences A3MZV4-11, A3MZV4-3.1 and A3MZV4-9 are from clonally related B cells and designated as clone set ^C#8^

^o^The sequences A3MZV4-20B and A3MZV4-7 are from clonally related B cells and designated as clone set ^C#9^

^p^The sequences A2RFV5-42 and A2MZV5-37 are from clonally related B cells and designated as clone set ^C#10^

Three of the four IGHV3 germline genes were expressed as productive genes in both B cell subsets, i.e. IGHV3S1, IGHV3S3 and IGHV3S5 (Table 1). From the IGHV4 gene family we were able to successfully amplify only one of the two potentially functional genes (viz. IGHV4S2). In total, we obtained 12 IGHV4-Cμ transcripts from FO-B cells and 59 IGHV4-Cμ transcripts from MZ-B cells (Table 1). From the second largest IGHV gene family in the BN rat, the IGHV5 gene family (also called the PC7183 family), 16 different, out of 26 potentially functional IGHV5 genes were found among 40 and 61 IGHV5-Cμ transcripts (see Table 1) that were amplified from FO-B cells and MZ-B cells, respectively.

### MZ-B cells express more mutated IGHV-Cμ transcripts than FO-B cells

**Fig 2:**
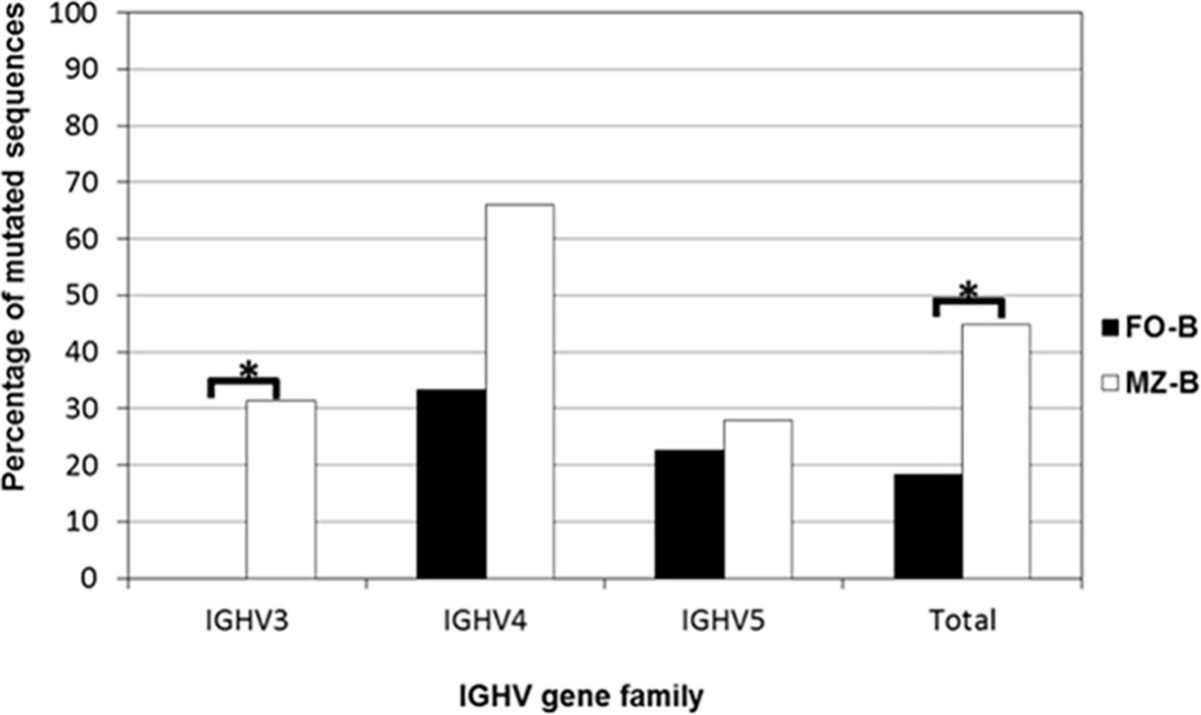
Percentage of mutated IgM^+^ FO-B cells and MZ-B cells within different IGHV gene families. Analysis of the proportion of mutated sequences (>2 mutations compared to the closest germline gene) shows that MZ-B cells express more mutated sequences than FO-B cells, when all sequences from the three IGHV gene families are combined (i.e. “total”) (Fisher’s exact test: P < 0.001). This difference between MZ-B cells and FO-B cells was largely due to a significant difference in the percentage of mutated sequences within the IGHV3 family (Fisher’s exact test: P = 0.016). There are relatively more mutated sequences found in the IGHV4 gene family compared to IGHV3 and IGHV5 both for the MZ-B cells (Fisher’s exact test: P < 0.001) and FO-B cells (Fisher’s exact test: P = 0.023).

We subsequently analyzed the obtained IGHV-Cμ transcripts that were amplified from both B cell subpopulations. The number of mutations within each rearranged IGHV gene was assessed on the basis of the nucleotide identity to the closest corresponding germline of the IGHV gene counterparts. Sequences with only 1 or 2 mutations were considered to be germline because we cannot exclude the possibility that these differences compared to germline IGHV genes were due to PCR artifacts [10]. We first analyzed the proportion of mutated sequences among the combined IGHV3, IGHV4 and IGHV5 sequences. As we show in Fig 2, 18% of FO-B cells and 45% of MZ-B cells expressed mutated IGHV-Cμ transcripts. This percentage of mutated sequences is significantly higher within the MZ-B cell subset compared to the percentage of mutated sequences present within the FO-B cell subset (Fisher’s exact test: P < 0,001).

### The difference in frequency of mutated sequences between MZ-B cells and FO-B cells is largely due to the IGHV3 gene family

We subsequently analyzed whether this difference in percentage of mutated sequences between FO-B cells and MZ-B cells was present in all three IGHV families tested. There was no statistical difference in percentage mutated sequences between the two B cell subsets among sequences of the IGHV4 and IGHV5 gene families, albeit that there was a strong trend with a higher percentage of MZ-B cells expressing mutated IGHV4 genes present compared to the percentage of mutated IGHV4 genes of FO-B cells (Fisher’s exact test: P = 0,051) (Fig 2). In contrast, within the IGHV3 gene family, 31% of the sequences derived from MZ-B cells were mutated, whereas none of IGHV3 sequences derived from FO-B cells were mutated (Fisher’s exact test: P = 0,016) (Fig 2). Thus, the higher frequency of MZ-B cells expressing mutated IGHV sequences is largely due to the contribution of the IGHV3 gene family.

### The IGHV4 gene family contains the highest percentage of mutated IGHV-Cμ transcripts, both among MZ-B cells and among FO-B cells

We analyzed whether there was a difference in the percentage of mutated IGHV-Cμ transcripts between the three IGHV gene families (IGHV3, IGHV4, IGHV5) in the two B cell subsets. As we show in Fig 2, within both MZ-B cells and FO-B cells, the IGHV4 gene family contained a significantly higher proportion of mutated IGHV-Cμ transcripts, compared to the two other IGHV gene families (IGHV3 and IGHV5) (Fisher’s exact test: P = 0,023 and P < 0,001, respectively). Of the IGHV4 sequences two-third of the MZ-B cell-derived sequences and one-third of the FO-B cell sequences were mutated. Thus, based upon these findings we conclude that there is a significant difference in the percentage of mutated sequences between the various IGHV gene families, for both MZ-B cells and FO-B cells.

### Mutation frequency among the mutated IGHV-Cμ transcripts is higher in MZ-B cells compared to FO-B cells

**Fig 3:**
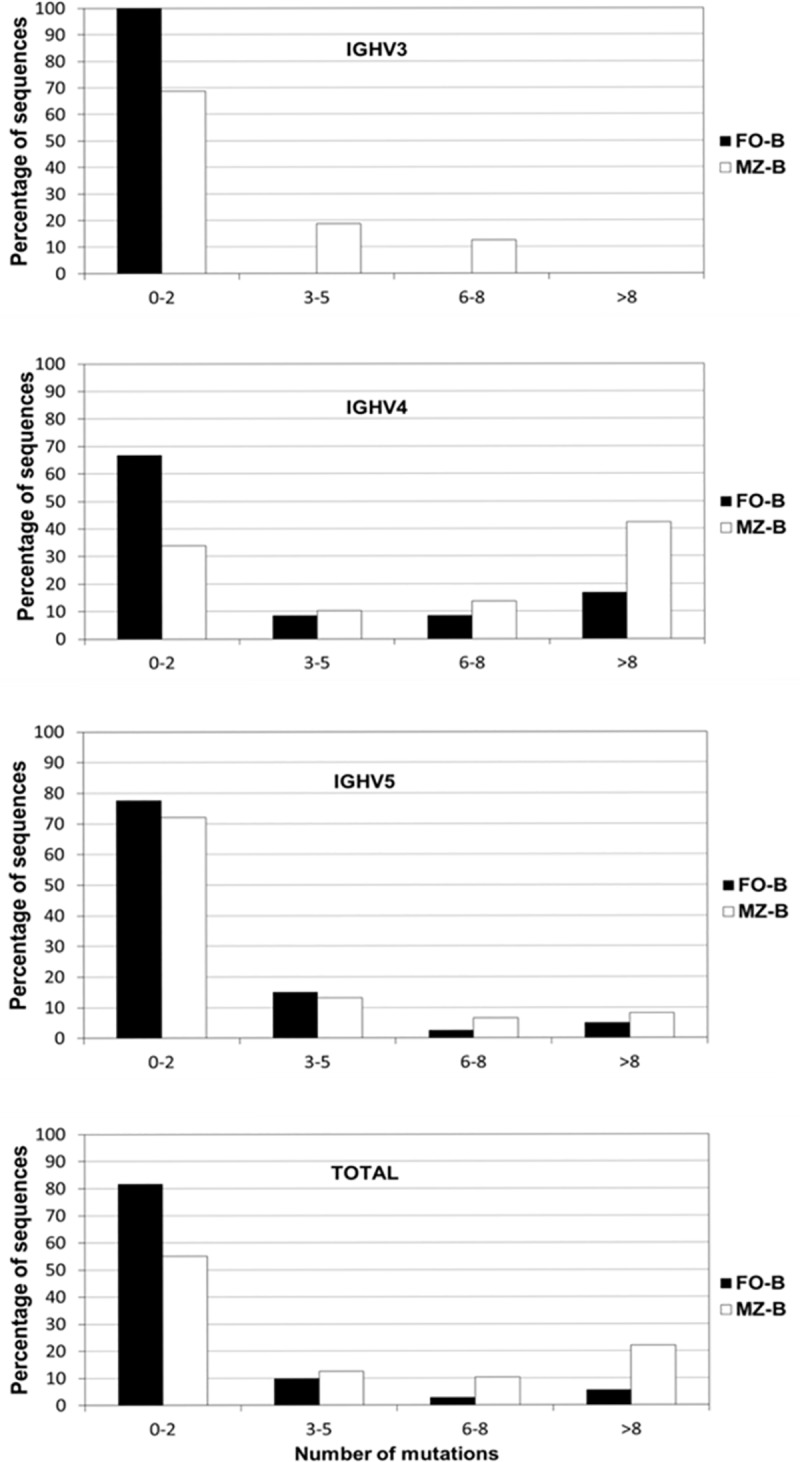
Mutation frequency of IGHV-Cμ transcripts of different IGHV families within MZ-B cells and FO-B cells. Analysis of the distribution of the number of mutations of all (“total”) IGH-Cμ transcripts shows that MZ-B cells have significantly more mutations per transcript than FO-B cells (Mann-Whitney P = 0.046). Within the MZ-B cell subset, IGHV4 sequences contained more mutations than in IGHV3 or IGHV5 sequences (Kruskal-Wallis, P = 0.011).

We next compared the number of mutations among the mutated IGHV-Cμ transcripts (i.e. > 2 mutations per transcript) IGHV genes in FO-B cells and MZ-B cells. When taking all mutated sequences from the three families together, MZ-B cells have a significantly (Mann-Whitney, P = 0.046) higher number of mutations than FO-B cells (Fig 3). In MZ-B cells, the number of mutations is 8.8±4.0 (median 8) and in FO B cells 6.8±3.9 (median 4). Further analysis revealed that the mutation frequency of mutated IGHV-Cμ sequences from MZ-B cells is significantly higher among IGHV4 sequences than among IGHV3 or IGHV5 sequences (Kruskal-Wallis, P = 0.011). These results indicate that the number of mutations in mutated MZ-B cells is higher in comparison to FO-B cells, which appears to be largely due to the higher number of mutations among the IGHV4 sequences (Fig 3).

### Clones of B cells are found within the MZ-B cell subset and some of these clones have members that are also present within the FO-B cell subset

H-CDR3 regions can be used to assess clonal relationships between B cells because the H-CDR3 region is virtually unique for each different IGHV rearrangement. A total of 10 independent clone sets (designated as C#1-C#10) were found among the two B cell subsets. Seven clone sets (2–3 members per clone) had members found exclusively found within the MZ-B cell subset and the remaining three clone sets had shared members between the MZ-B cell subset and the FO-B cell subset (see Table 1). The sequences that belonged to clonally related cells with 2 or 3 members only found in the MZ-B cell subset included clone sets using IGHV3: clone set C#1 (A2MZV3-B7, A2MZV3-F5), clone set C#2 (A2MZV3-C6, A2MZV3-C7, A2MZV3-E7), clone set C#8 (A3MZV4-11, A3MZV4-3.1, A3MZV4-9) and clone set C#9 (A3MZV4-20B, A3MZV4-7) or clone sets using IGHV4: clone set C#5 (IGA2MZV4-14, A2MZV4-3), clone set C#6 (A2MZV4-2.13, A2MZV4-2.17) and clone set C#7 (A2MZV4-2.9, A2MZV4-7). IGHV genes used by members of clone sets C#1 and C#2 had none or only one mutation in their IGHV genes. Members from other clone sets (C#5,6,8 and 9) exhibited more mutations (more than 6 mutations per IGHV sequence); most of these mutations were shared between the members of a clone. Sequences from three clone sets C#3 (A2RFV4-2, A2MZV4-1), C#10 (A2RFV5-42, A2MZV5-37) and C#4 (A3RFV4-3, A3MZV4) have members found in both B cell subsets. Clone sets C#3 and C#10 exhibited an identical mutation pattern, while clone set C#4 showed many shared mutations. Overall, most of these clonally related sequences thus displayed both shared mutations in combination with unique mutations, indicating that members from one clone set were probably derived from the same naive precursor cell.

### Analysis of the neonatal IGHV genes in FO-B cells and MZ-B cells

**Fig 4:**
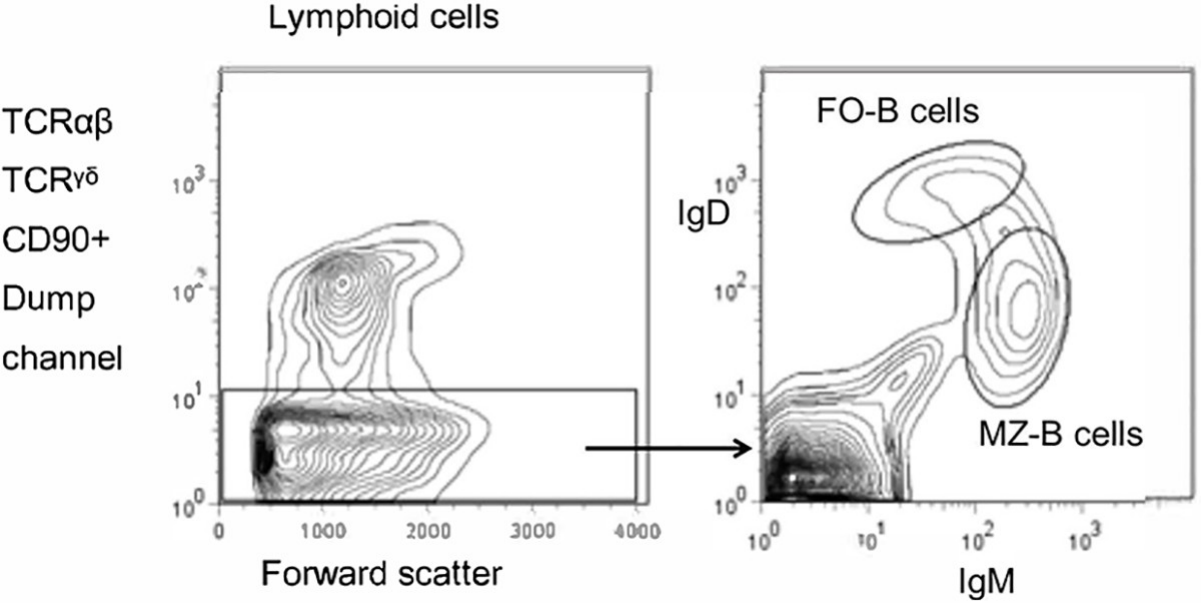
Three-colour cytometry was used to analyze FO-B cells and MZ-B cells. **A** single neonatal rat splenic cell suspension was stained with FITC conjugated anti-rat IgM (HIS40; eBioscience, San Diego, CA, USA), biotinylated anti-rat IgD (MaRD3; AbD Serotec, Oxford, UK), and APC anti-rat CD90/Thy1.1 (HIS51; eBioscience). Biotinylated mAb were revealed with streptavidin conjugated to the tandem fluorochrome PE-Cy5.5 (Ebioscience). Lymphocytes were sequentially gated by forward scatter and side scatter. Acquisition gates were set to exclude the unwanted immature B cells (CD90^+^-APC). Gate settings were set appropriately for FO-B cells (CD90^neg^IgD^high^IgM^low^) and MZ-B (CD90^neg^IgM^high^IgD^low^) cells.

Weller et al and others postulated that mutations in MZ-B cells are not the consequence of an antigen-driven response, but are an intrinsic property of these B cells, introduced during their development [25, 26]. In this study we also addressed this issue, by analysing the occurrence of SHM in IGHV genes, expressed by MZ-B cells present in neonatal rats, i.e. at a time point when antigen-driven humoral immune responses have not been established, as witnessed by the absence of GC in lymphoid organs during the first few weeks of life [33, 34]. MZ-B cells and FO-B cells were sorted from (pooled) neonatal rat spleens, as is illustrated in Fig 4. A large proportion of MZ-B cells in neonatal rats express CD90 and are therefore considered to represent immature MZ-B cells [10]; mature MZ-B cells and FO-B cells are defined as CD90^-^IgM^high^IgD^low^ cells and CD90^-^IgM^low^IgD^high^ respectively. By including CD90 in our staining combination, we excluded immature MZ-B and FO-B cells from our analysis.

In the analysis of adult IGHV genes, we showed that the proportion of mutated IgM^+^ MZ-B cells varied significantly when different IGHV gene families were analyzed. This proportion of mutated MZ-B cell-derived sequences occurred up to 28% for IGHV5 transcripts and up to 66% for IGHV4 transcripts in adult rats. For this reason, we chose to analyze IGHV-Cμ transcripts encoding for IGHV5 and IGHV4 family genes. IGHV-Cμ mRNA transcripts from both neonatal FO-B cells and MZ-B cells were amplified by RT-PCR, cloned, sequenced and analyzed for the presence of SHM. The IGHV5 gene family is the second largest IGHV gene family in the rat and consists of 27 potentially functional IGHV genes [37]. In total, we amplified 16 unique IGHV5-Cμ transcripts from neonatal MZ-B cells and 22 IGHV5-Cμ transcripts from neonatal FO-B cells (Table 2). Various germline genes encoded these transcripts: 10 different IGHV5 germline genes were used by MZ-B cells and 12 by FO-B cells. The IGHV4 gene family is composed of only 2 potentially functional IGHV genes [37], of which only one (IGHV4S2) seemed to be expressed. We obtained 21 unique IGHV4-Cμ sequences derived from neonatal MZ-B cells and nine IGHV4-Cμ sequences from neonatal FO-B cells (Table 2). The IGHV4S2 gene encoded all these transcripts, confirming our previous findings in adult rats, that only this family member is functionally expressed by rat B cells. As shown in (Table 2), the mutational analysis of the IGHV5-Cμ and IGHV4-Cμ transcripts revealed that none of the transcripts from either MZ-B cells or FO-B cells were mutated (i.e. expressed more than two nucleotide differences compared to their germline counterparts). Since GC are absent in the first few weeks of neonatal rats [34, 38], the finding that mutated IgM^+^ memory B cells are absent in neonatal animals supports the hypothesis that IgM^+^ memory B cells are generated in the GCs. The absence of mutation profiles of IGHV genes in neonatal rats therefore revealed that mutated IgM MZ-B cells are possibly generated in an antigen GC-dependent process in rats as opposed to the GC-independent process proposed by Weller et al. for humans [25].

**Table 2:** Neonatal IGHV Cμ mRNA transcripts from FO-B cells and MZ-B cells.

**Clone**^**a**^	**IGHV**	**IGHD**	**IGHJ**	**Mutations**^**b**^	**H-CDR3**^**c**^	
** **	**member**	**member**	**member**	** **	**N**^**f**^	**Amino acid**
**Sequences of IGHV4 gene family from FO-B**^**d**^ **cells**						
NRFVH4-60	IGHV4S2	IGHD5-1	IGHJ2	0	7	ATGSFDY
NRFVH4-71^g^	IGHV4S2	IGHD1-6	IGHJ2	1	9	ARAPGGYDY ^C#1^
NRFVH4-16	IGHV4S2	IGHD1-1	IGHJ2	0	14	ARESYYYYSGDFDY
NRFVH4-37^g^	IGHV4S2	IGHD1-6	IGHJ2	1	9	ARAPGGYDY^C#1^
NRFVH4-2.1	IGHV4S2	IGHD1-6	IGHJ2	0	11	ARAGGYYYFDY
NRFVH4-2.4	IGHV4S2	IGHD1-2	IGHJ2	0	10	ARVLWVYFDY
**Clone**^**a**^	**IGHV**	**IGHD**	**IGHJ**	**Mutations**^**b**^	**H-CDR3**^**c**^	
** **	**member**	**member**	**member**	** **	**N**^**f**^	**Amino acids**
NRFVH4-2.5	IGHV4S2	IGHD1-4	IGHJ2	0	12	ARAYYGYNYFDY
NRFVH4-2.6	IGHV4S2	IGHD1-4	IGHJ2	0	11	ARYYGYNYFDY
NRFVH4-2.7	IGHV4S2	IGHD1-4	IGHJ2	0	12	ATYYGYNYYFDY
**Sequences of IGHV4 gene family from MZ-B**^**e**^ **cells**						
NMZVH4-1	IGHV4S2	IGHD1-1	IGHJ1	0	15	AIMYTTDYXYWYFDF
NMZVH4-3	IGHV4S2	IGHD1-7	IGHJ2	0	11	ARAYYDGSYYY
NMZVH4-4	IGHV4S2	IGHD1-1	IGHJ4	0	16	ARAHMYTTDYYYVMDA
NMZVH4-5	IGHV4S2	IGHD1-5	IGHJ1	0	10	AIYNNWYFDF
NMZVH4-6	IGHV4S2	IGHD1-4	IGHJ2	0	10	ARLPGYNFDY
NMZVH4-9	IGHV4S2	IGHD2-2	IGHJ2	0	9	ARDTYYFDY
NMZVH4-13	IGHV4S2	IGHD1-3	IGHJ1	0	12	ARARSSYWYFDF
NMZVH4-17	IGHV4S2	IGHD1-7	IGHJ2	0	12	ARNYPGMYYFDY
NMZVH4-20	IGHV4S2	IGHD1-6	IGHJ2	0	8	ARTEGIDY
NMZVH4-3.1	IGHV4S2	IGHD3-2	IGHJ2	0	10	ARARYNYFDY
NMZVH4-3.2^h^	IGHV4S2	IGHD1-7	IGHJ1	1	16	ARFYYDGSYYYWYFDF^C#2^
NMZVH4-3.3	IGHV4S2	IGHD1-3	IGHJ2	0	10	ARYSSYYFDY
NMZVH4-3.4	IGHV4S2	IGHD5-1	IGHJ2	0	8	ATGSYFDY
NMZVH4-3.6^h^	IGHV4S2	IGHD1-7	IGHJ1	0	16	ARDYYDGSYYYWYFDF^C#2^
NMZVH4-3.7	IGHV4S2	IGHD1-3	IGHJ2	0	6	ARGSYY
NMZVH4-3.8	IGHV4S2	IGHD4-1	IGHJ2	0	9	ARAQFGVDY
NMZVH4-3.9	IGHV4S2	IGHD1-5	IGHJ2	0	9	ARIYNNFDY
NMZVH4-3.10	IGHV4S2	IGHD5-1	IGHJ2	1	11	ARTGYYWSFDF
NMZVH4-3.11	IGHV4S2	IGHD5-1	IGHJ2	1	10	ARDWELYFDY
NMZVH4-3.12	IGHV4S2	IGHD5-1	IGHJ1	0	12	ARTGSYYWYFDF
NMZVH4-3.13	IGHV4S2	IGHD1-4	IGHJ2	0	13	ARRYYGYNYYFDY
**Sequences of IGHV5 gene family from FO-B**^**d**^ **cells**						
NRFVH5-50	IGHV5S30	IGHD4-1	IGHJ1	1	13	ATDNSGYYWYFDF
NRFVH5-72	IGHV5S30	IGHD1-4	IGHJ2	0	13	ATIAAISTYYFDY
NRFVH5-8	IGHV5S30	IGHD5-1	IGHJ2	0	8	ATGSYFDY
NRFVH5-18	IGHV5S30	IGHD1-2	IGHJ3	1	9	ATGYNWFAY
NRFVH5-1	IGHV5S27	IGHD1-7	IGHJ2	0	12	ARHYYSGDYFDY
NRFVH5-3	IGHV5S13	IGHD1-8	IGHJ2	0	14	ARHYYDGYYHYFDY
NRFVH5-4	IGHV5S11	IGHD4-1	IGHJ3	1	12	ARHNSGYNWFAY
NRFVH5-6	IGHV5-6	IGHD1-2	IGHJ2	0	8	TTDHYGDY
NRFVH5-8	IGHV5S27	IGHD1-7	IGHJ2	0	14	ARHYYDGSYYYFDY
NRFVH5-9	IGHV5S10	IGHD1-5	IGHJ1	2	12	ATHNNYYWYFDF
NRFVH5-10	IGHV5-1	IGHD1-3	IGHJ2	0	11	ANYYYSSYIDY
NRFVH5-11	IGHV5S74	IGHD1-1	IGHJ3	1	12	ARMYTTDNWFAY
NRFVH5-12	IGHV5S10	IGHD1-8	IGHJ2	0	10	ATHYYDGYYY
**Clone**^**a**^	**IGHV**	**IGHD**	**IGHJ**	**Mutations**^**b**^	**H-CDR3**^**c**^	
** **	**member**	**member**	**member**	** **	**N**^**f**^	**Amino acids**
NRFVH5-13	IGHV5-6	IGHD4-1	IGHJ2	0	10	TTNSGYYFDY
NRFVH5-14	IGHV5S57	IGHD1-8	IGHJ2	1	12	TNYRDSYAYFDY
NRFVH5-15	IGHV5S45	IGHD1-6	IGHJ2	1	10	ARQLRRVFDY
NRFVH5-16	IGHV5S10	IGHD1-6	IGHJ3	0	10	ATYGGYWFAY
NRFVH5-17	IGHV5S57	no results	IGHJ3	0	8	TRGYWFAY
NRFVH5-18	IGHV5S29	IGHD1-7	IGHJ2	0	15	TTETYYYDGSYYFDY
NRFVH5-19	IGHV5S16	IGHD5-1	IGHJ1	0	9	ARGSWYFDF
NRFVH5-2.3	IGHV5S30	IGHD4-1	IGHJ2	0	11	ATDNSGYYFDY
**Sequences from IGHV5 gene family from MZ-B**^**e**^ **cells**						
NMZVH5-2	IGHV5S30	no results	IGHJ2	0	4	ATNY
NMZVH5-10	IGHV5S30	IGHD4-1	IGHJ2	0	10	ATDSGYYFDY
NMZVH5-2	IGHV5-3	IGHD1-2	IGHJ4	0	10	ARHGYYVMDA
NMZVH5-3	IGHV5-2	IGHD1-8	IGHJ2	0	10	ARHDGYYFDY
NMZVH5-5	IGHV5S43	IGHD1-5	IGHJ2	0	10	TRDNNYYFDY
NMZVH5-6	IGHV5S65	no results	IGHJ2	1	8	AKAHYFDY
NMZVH5-11	IGHV5S10	IGHD1-4	IGHJ2	1	11	ATHYGYNYFDY
NMZVH5-12	IGHV5-6	IGHD1-7	IGHJ2	1	12	ARHYYDGSYYDY
NMZVH5-14	IGHV5S32	IGHD1-7	IGHJ2	1	14	ARHYDGSYYYYFDY
NMZVH5-15	IGHV5S16	IGHD4-1	IGHJ3	0	9	ARHNSGFAY
NMZVH5-16	IGHV5S16	IGHD1-4	IGHJ2	0	6	ATHNDY
NMZVH5-17	IGHV5S30	IGHD1-3	IGHJ4	0	10	ATYSSYVMDA
NMZVH5-18	IGHV5S43	IGHD1-5	IGHJ2	0	11	TRDHNNYYFDY
NMZVH5-20	IGHV5S11	IGHD1-6	IGHJ1	0	16	ARHNYGGYSDYWYFDF
NMZVH5-3.15	IGHV5S30	IGHD1-3	IGHJ2	0	8	ATEYWSDY
NMZVH5-3.16	IGHV5S30	IGHD5-1	IGHJ2	0	9	ATTGSYFDY

^a^Cμ (IgM) transcripts from FO-B cells and MZ-B cells

^b^ Mutations, nucleotide differences of one or more basis between IMGT germline gene and rearranged Cμ transcript

^c^H-CDR3, heavy chain complementarity determining region 3

^d^FO-B cells, recirculating follicular B cells

^e^MZ-B cells, marginal zone B cells

^f^Lenght of H-CDR3 in amino acids

^g^ The sequence NRFVH4-71 and NRFVH4-37 are from clonally related B cells and designated as clone set ^C#1^

^h^ The sequence NMZVH4-3.2^h^ and NMZVH4-3.6^h^ are from clonally related B cells and designated as clone set ^C#2^

## Discussion

Previous studies provided evidence supporting the existence of mutated, IgM^+^ expressing, memory MZ-B cells in the rat [39]. Dammers et al. [40] demonstrated that less than 20% of the MZ-B cells isolated from spleens of PVG rats carried mutated IGHV genes. These findings were in marked contrast to humans, where >95% of the splenic MZ-B cells are mutated [22, 41, 42]. One possible explanation for this difference could be that only one particular IGHV gene family (viz. the IGHV5 family, the homolog of PC7183 in the mouse) was analyzed in the (PVG) rat and that this IGHV gene family was not representative of other IGHV genes, or IGHV gene families. By establishing the genomic germline IGHV gene repertoire of the BN rat [37] it became possible to accurately analyze other IGHV gene families as well. To avoid possible strain differences we used the BN rat strain, rather than the PVG rat strain that was used previously [39]. Here we report on the frequency of mutated sequences in rearranged IGHV-Cμ transcripts derived from FACS-sorted MZ-B cells (IgM^high^IgD^low^) in comparison with FO-B cells (IgM^low^IgD^high^) obtained from the adult BN rat spleen. The analysis was confined to three different IGHV gene families, which differ in size: IGHV3, IGHV4 and IGHV5. These three IGHV gene families have 4, 2 and 26 functional IGHV genes, respectively. The IGHV3 and IGHV4 gene families were chosen to determine whether there is a difference in mutation frequencies among members of the IGHV gene families that are relatively small and to compare this frequency to the second largest IGHV gene family (IGHV5) in the rat, which had been analyzed previously in the PVG rat [10]. The BN rat strain contains 26 functional IGHV5 (germline) genes compared to the 28 germline genes of the PVG rat. In agreement with previous publications [41, 43, 44], we found that splenic MZ-B cells express a significantly higher percentage of mutated sequences than FO-B cells and all three analyzed IGHV gene families contributed to this difference. In BN rats a slightly higher proportion (27%) of the MZ-B cells expressed mutated IgM molecules encoded by IGHV5 family genes, compared to this proportion in the PVG rat (10–20%) [10]. This difference in mutation frequency can be due to differences that exist between the PVG and BN rat strains, such as for example the fact that BN rats have fewer IGHV genes, or it might also be caused by different environmental conditions (microbial environment; different microbiota) of the two rat strains. Analysis of the IGHV3 gene family showed that a similar proportion (approximately 30%) of mutated IgM encoding sequences can be found among the BN rat-derived MZ-B cells. In marked contrast, to these two IGHV families, a high proportion (66%) of the IGHV4 sequences from purified MZ-B cells was mutated. This family consists of only two potentially functional IGHV genes, although only one of these appeared to be functionally expressed. The presented findings show that the proportion of mutated sequences derived from MZ-B cells varies between the different IGHV gene families in the BN rat. In total a higher proportion (27–66%) of IGHV genes was mutated compared to the 10–20% of mutated sequences found previously for the IGHV5 gene family in PVG rats [10, 20]. Our observation that the highest percentage of mutated frequencies occurred in the single functional member IGHV4 gene family, suggests that there could be more antigen selection pressure on this particular IGHV4 gene in expanding its available repertoire by SHM. Although in total a higher average number of mutated sequences was observed among rat MZ-B cells was observed than previously, the frequency of mutated sequences among human MZ-B cells is still much higher. In humans, nearly all MZ-B cells are mutated [22, 41, 42]. Since the analysis of mutated IGHV genes was restricted to a particular set of IGHV genes in humans, the observed difference in frequency of mutated IGHV genes between the different IGHV families may also contribute to the reported difference in incidence of mutated MZ-B cells in humans and rats. Dunn-Walters et al. [45] analyzed only two particular IGHV genes: the IGHV6 gene and IGHV4.21 gene. It is possible that these IGHV genes are more mutated than other genes. However the analysis of Tangye et al. [42] showed that Ig genes isolated from IgM^+^ memory B cells among IGHV5 and IGHV6 gene families were all mutated which shows that the high frequency of mutations is not limited due to individual IGHV genes. Further, Colombo et al. [41] found that most of the human IGHV1, IGHV3 and IGHV4 gene families among splenic-derived MZ-B cells (IgM^high^CD27^+^), GC B cells and class-switched B cells were mutated, albeit with a lower average number of mutations than both GC and class-switched B cells. However, the average number of mutations in human MZ-B cells (11.8) [41] was higher than both IgM^+^ MZ-B cells (8.8) and IgG^+^ MZ-B cells (7) [21] found in rats. This might be because humans have fewer functional IGHV genes than rats. We postulate that the higher number of germline IGHV genes in rodents is due to rats requiring fewer mutations to diversify their antibody repertoire after immunization than humans, because rats can encode a larger pool of different antibodies from their primary repertoire. In addition, it is possible that differences in life span and environmental conditions also contribute to differences in the average mutation frequency per IGHV gene. During their long lives, humans may encounter a greater variety of antigens than laboratory rats that live in well-controlled laboratory conditions.

Several pathways for the development of IgM and IgG B cells have been proposed. As previously suggested [46], shorter H-CDR3 regions of the IgM molecules expressed by naïve MZ-B cells in rats [40] and mice [8] are associated with polyreactive antibody responses and are ligand selected to bind to TI-antigens such as carbohydrates of micro-organisms [47]. Panda and Ding [48] proposed that splenic MZ-B cells such as B-1 cells might also be involved in the secretion of natural IgM and IgG antibodies. The authors went on to propose that natural antibodies, in particular IgM with diverse immune functions, could link the innate to the adaptive immune system. Findings of earlier experimental studies support these notions, revealing that natural IgM antibodies can recognize foreign antigens such as phosphorylcholine and modify low-density lipoprotein antigens [49]. Indeed IgM antibody production against both TI and TD antigens are induce by neutrophils activating MZ-B cells via BAFF, APRIL and IL-21. In addition to unmutated IgM molecules our data directly showing the presence of mutated IGHV-Cμ transcripts among the pool of purified rat IgM^high^IgD^low^ MZ-B cells.

Memory cells are generally believed to be generated in GCs. It is still controversial, however, whether mutated (memory) IgM^+^ MZ-B cells are derived from GCs or whether they represent a GC-independent B cell population. Among the mutated sequences, we observed groups of mutated sequences that were derived from clonally related B cells, i.e. these sequences had identical H-CDR3 regions, used the same IGHV gene, expressed shared mutations and were from the same rat. Most (70%) of groups of cells had members that were confined to the MZ-B cell compartment. However, importantly, some clonally related groups of cells had members that were found among both MZ-B cells and FO-B cells. This clonal relationship strongly suggests that mutated IgM^+^ MZ-B cells and mutated IgM^+^ FO-B cells have a common origin. This is consistent with our previous finding that clonally related class-switched, mutated B cells co-exist with members in both the MZ-B and FO-B cell compartment [21]. Possibly both unswitched and class-switched B cells are generated in the same fashion. Somatic hypermutations (SHM) are usually introduced in B cells proliferating in the GC environment. Mutated B cells are subsequently subjected to some form of positive selection for B cells expressing immunoglobulins that bind with high affinity to antigen presented by follicular dendritic cells. Genetically engineered mice (such as Bcl6 deficient or CD40 deficient mice) cannot form GCs and lack B cells with mutated IGHV genes, including mutated IgM genes [50, 51]. Thus, at least in mice, GCs appear to be critically involved in SHM of IGHV genes during regular immune responses. This indicates that both mutated IgM^+^ FO-B cells and MZ-B cells are probably GC derived. In contrast to mice, mutated IgM^+^ B cells can still be found in humans with CD40 or CD40L deficiency (hyper IgM syndrome patients, HIGM), that lack classical CD40L mediated T cell help and lack GC formation [30, 52–54]. These mutated B cells are IgM^+^IgD^+^CD27^+^ cells, also called natural effector cells, that correspond to splenic MZ-B cells [54]. Other CD27^+^ B cell populations could not be formed in CD40/CD40L deficient HIGM patients. Berkowska et al. [53] observed that IgM^+^IgD^+^CD27^+^ cells have a relatively low replication history. Furthermore they are already present in very young (< 2 years) children and in human foetuses [26, 31, 54]. These findings suggest that at least a significant proportion of these mutated IgM^+^IgD^+^CD27^+^ MZ-B (-like) cells are not derived from GCs and are generated in the absence of T cell help. Weill and colleagues [54–56] speculated that these MZ-B (-like) cells use SHM in order to diversify their repertoire early during ontogeny outside T-dependent or T-independent humoral immune responses. However, this hypothesis has been challenged in the literature [27, 28]. Our observation that there are clonally related FO-B cells and MZ-B cells expressing mutated IgM molecules indicates that these cells have a common origin and provide evidence that does not support the notion that such a postulated diversification process would then be unique to MZ-B cells. In support of this, the variation in the percentage of mutated sequences in the various IGHV gene families also showed that SHM is used for diversification. In the case of pre-diversification it would be more likely that mutations occur more or less at a similar rate in all IGHV gene families. This variation in mutation frequency between different IGHV gene families is more in favour of a diversification because of antigenic stimulation. In humans, Colombo et al. [41] observed a small number of clonally related sequences that were shared between MZ-B cells and GC B cells, indicating that mutated IgM^+^ MZ-B cells can be derived from GCs. Recently, Aranburu and co-workers [57] proposed that three different populations of IgM memory B cells exist in humans and that most IgM memory B cells develop independently of the GC. These data suggest that during the first stages of life (i.e. 6–7 years), these cells can enter a GC to become either “remodeled” IgM memory B cells or class-switched memory B cells. In contrast, Weill and colleagues [26, 54, 56] suggested that the mutated IgM^+^ MZ-B cells are not GC-derived memory B cells. Instead, these authors postulated that the mutations in human MZ-B cells are acquired during their development in order to diversity their primary repertoire, in a GC and T-cell independent fashion. To test this hypothesis in rats we investigated the possible presence of mutated IgM^+^ MZ-B cells in neonatal rats. Neonatal rats do not develop GCs in the first weeks of life [34, 38]. Thus, when MZ-B cells are unmutated in neonatal rats this would strongly argue against the hypothesis of Weill et al, [25, 26, 56], that SHM is part of the developmental program of MZ-B cells. To this end, we analyzed IGHV-Cμ transcripts using IGHV4 and IGHV5 gene families from both MZ-B cells and FO-B cells. In summary, no mutations were found in any of the neonatal sequences, not even in the IGHV4 gene family genes that have the highest number of mutated sequences (66%) in the adult rat. These results support the notion that at least in rats, the mutated IgM^+^ MZ-B cells seen in adult animals are bona fide memory cells which are most probably generated under the influence of external antigenic stimuli in the GC.

## Abbreviations

FO-B: follicular B
GC: germinal centre
IGHV: immunoglobulin heavy chain variable region
H-CDR3: immunoglobulin heavy chain complementarity determining region 3
SHM: somatic hypermutation
MZ-B: marginal zone B cell
TI-2: T cell-independent type 2 antigens
